# Elucidating the causal relationship of mechanical power and lung injury: a dynamic approach to ventilator management

**DOI:** 10.1186/s40635-025-00736-w

**Published:** 2025-02-28

**Authors:** ChaoPing Wu, Arif Canakoglu, Jacob Vine, Anya Mathur, Ronit Nath, Markos Kashiouris, Piyush Mathur, Ari Ercole, Paul Elbers, Abhijit Duggal, Ken Koon Wong, Anirban Bhattacharyya

**Affiliations:** 1 Critical Care, Integrated Hospital Care Institute, Cleveland Clinic, 9500 Euclid Avenue, Cleveland, OH 44195 USA; 2 Department of Anestesia, Intensive Care and Emergency, Fondazione IRCCS Ca’ Granda Ospedale Maggiore Policlinico, Via Francesco Sforza 35, 20122 Milan, Italy; 3 Center for Resuscitation Science, Beth Israel Deaconess Medical Center, 1 Deaconess Rd, Boston, MA 02215 USA; 4 Western Reserve Academy, 115 College St, Hudson, OH 44236 USA; 5 Computer Science, University of California, Berkeley, 387 Soda Hall, Berkeley, CA 94720 USA; 6 Critical Care, INOVA Fairfax Hospital, 3300 Gallows Rd, Falls Church, VA 22042 USA; 7 Anesthesiology, Integrated Hospital Care Institute, Cleveland Clinic, 9500 Euclid Avenue, Cleveland, OH 44195 USA; 8 Cambridge Center for Artificial Intelligence in Medicine., 3rd Floor University Centre, Granta Pl, Mill Lane, Cambridge, CB2 1RU UK; 9 Cambridge University Hospitals, NHS Foundation Trust, Hills Road, Cambridge, Cambridgeshire, CB2 0QQ UK; 10 Intensive Care Medicine, Amsterdam UMC, Meibergdreef 9, 1105 AZ Amsterdam, Netherlands; 11 Infectious Diseases, Integrated Hospital Care Institute, Cleveland Clinic, 9500 Euclid Avenue, Cleveland, OH 44195 USA; 12 Critical Care, Mayo Clinic, 4500 San Pablo Rd S, Jacksonville, FL 32224 USA

**Keywords:** Mechanical power, Mechanical ventilation, Causal inference, Counterfactual testing, Machine learning, Direct acyclic graph, Outcomes research

## Abstract

**Background:**

Mechanical power (MP) serves as a crucial predictive indicator for ventilator-induced lung injury and plays a pivotal role in tailoring the management of mechanical ventilation. However, its application across different diseases and stages remains nuanced.

**Methods:**

Using AmsterdamUMCdb, we conducted a retrospective study to analyze the causal relationship between MP and outcomes of invasive mechanical ventilation, specifically SpO_2_/FiO_2_ ratio (P/F) and ventilator-free days at day 28 (VFD28). We employed causal inferential analysis with backdoor linear regression and double machine learning, guided by directed acyclic graphs, to estimate the average treatment effect (ATE) in the whole population and conditional average treatment effect (CATE) in the individual cohort. Additionally, to enhance interpretability and identify MP thresholds, we conducted a simulation analysis.

**Results:**

In the study, we included 11,110 unique admissions into analysis, of which 58.3% (6391) were surgical admissions. We revealed a negative and significant causal effect of median MP on VFD28, with estimated ATEs of −0.135 (95% confidence interval [CI]: −0.15 to −0.121). The similar effect was not observed in Maximal MP and minimal MP. The effect of MP was more pronounced in the medical subgroup, with a CATE of −0.173 (95% CI: −0.197 to −0.143) determined through backdoor linear regression. Patients with cardio, respiratory, and infection diagnoses, who required long-term intubation, sustained higher impact on CATEs across various admission diagnoses. Our simulations showed that there is no single MP threshold that can be applied to all patients, as the optimal threshold varies depending on the patient’s condition.

**Conclusion:**

Our study underscores the importance of tailoring MP adjustments on an individualized basis in ventilator management. This approach opens up new avenues for personalized treatment strategies and provides fresh insights into the real-time impact of MP in diverse clinical scenarios. It highlights the significance of median MP while acknowledging the absence of universally applicable thresholds.

**Supplementary Information:**

The online version contains supplementary material available at 10.1186/s40635-025-00736-w.

## Background

Mechanical power (MP) is a predictive indicator for ventilator-induced lung injury (VILI) and represents a more comprehensive assessment of stress and increase sensitivity to ventilation changes compared to pressure or energy alone [1]. Previous studies demonstrated elevated MP can lead to lung damage even when pressure is low [2, 3]. In clinical practice, MP can be used to evaluate disease severity and optimize ventilator settings in patients with critical illness.

Despite its increasing recognition, the precise role of MP, along with its variability across diseases and disease stages, remains uncertain [4]. Whist MP is more complex than simple pressure and volume, it is readily computable from ventilator data.

Patient with heterogenous lung characteristics requires individualized MP consideration. Recent studies have underscored the variability in MP values among patients with different pulmonary pathologies such as acute respiratory distress syndrome (ARDS) and chronic obstructive pulmonary disease (COPD) [5]. Furthermore, even within the same disease, MP requirements can evolve with disease progression, as seen in a longitudinal study involving ARDS patients [6].

Individualized MP in heterogenous critically ill patients requires a comprehensive approach that considers patient comorbidities, disease characteristics, and clinical course [7]. Randomize controlled trials (RCTs) are the gold standard for determining causality [8], but they are difficult to conduct in critically ill patients, and their results can be difficult to generalize due to heterogeneity across geographical areas, sites, and time-varying clinical thresholds [9]. On the other hand, machine learning models can provide accurate prediction based on existing data. However, they are susceptible to bias due to unobserved variables and data drift, which make the result difficult to be generalized [10, 11]. Data-driven causal inferential analysis is a promising approach for individualized MP in critically ill patients. It surpasses classic predictive machine learning and multivariate logistic regression by not only isolating treatment impact, but also in yielding probabilistic outcome post-treatment [12, 13]. This approach facilitates the inference of the impact of MP adjustments on patient outcome while accounting for confounding variates.

Through the utilization of causal inference, our objective is to estimate the impact of MP on patients with invasive mechanical ventilators (IMV) within intricate real-world clinical environments using a causal inference model using the Amsterdam University Medical Centers Database (AmsterdamUMCdb). This approach holds the potential to unveil the influence of MP across heterogenic populations and disease stages in patients with critical illness.

## Methods

### Study setting

In this retrospective study, we use AmsterdamUMCdb which contains de-identified patient information on demographics, ventilator settings, vital signs, laboratory tests, and medications. It is also linked to clinical outcomes data, such as length of stay in the ICU and mortality [14].

The study included adult (≥18 years old) patients who underwent IMV during hospitalization. Exclusion criteria included incomplete data required to calculate MP, and patients under any spontaneous ventilation mode. Sub-cohorts were defined based on the specific intensive care units and admission reasons, including surgical reasons such as cardiovascular surgery and neurosurgery, as well as medical reasons like respiratory failure and cardiac arrest (Supplemental Fig. 1).

**Fig. 1:**
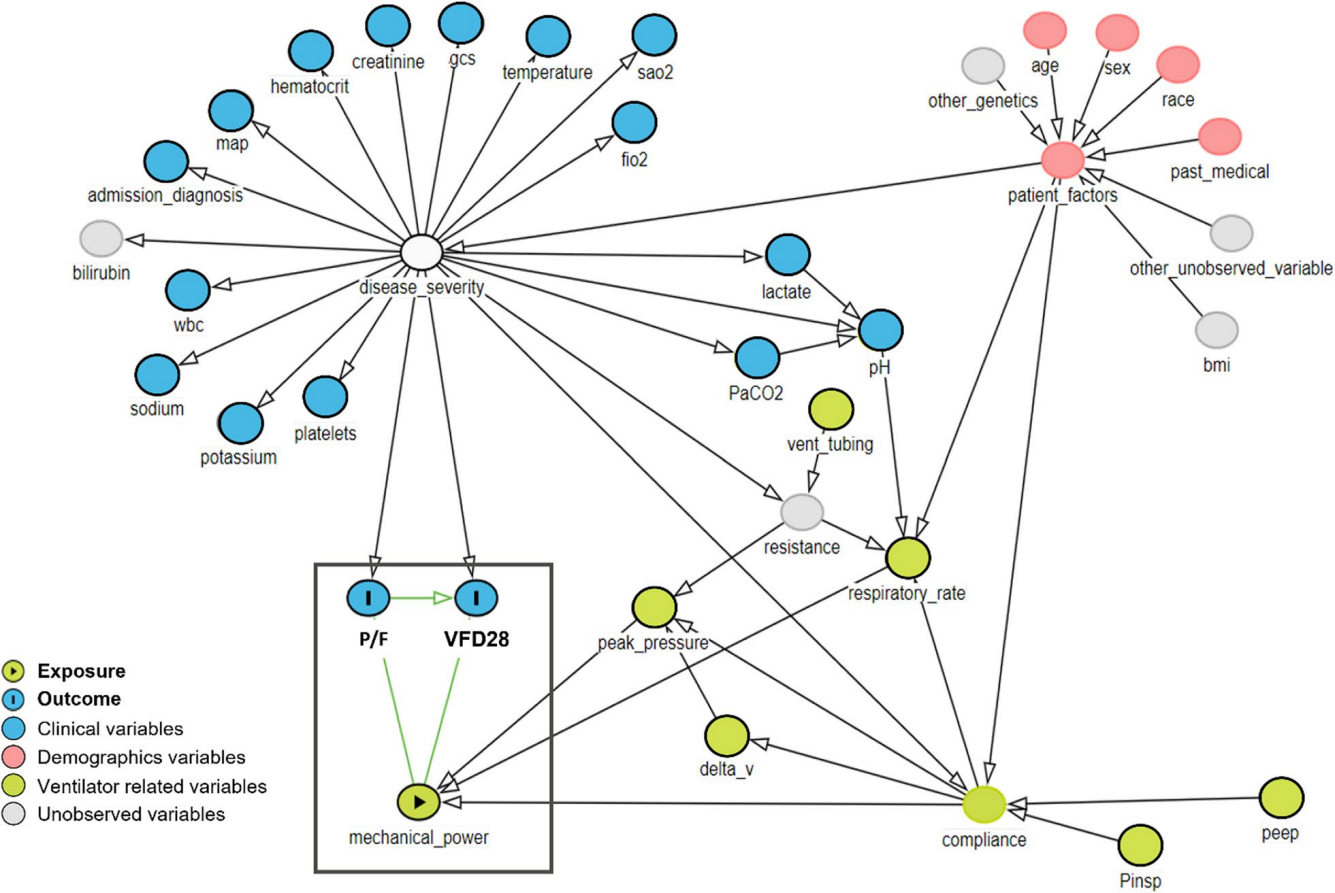
A directed acyclic graph (DAG) is employed to examine the causal effect of mechanical power on a clinically relevant outcome ventilator-free days through an intermediary outcome of PaO_2_/FiO_2_ ratio (represented by nodes with “I”). This DAG is constructed using clinical expertise. The *blue nodes* encompass relevant clinical variables, the pink nodes encompass patient demographic information, and the green nodes encompass invasive mechanical ventilator settings

### Exposure and outcome measure

The primary exposure in the study was the mechanical power (MP), defined as follows:

MP (J/min) = 0.098 × *V*_t_ × RR × (PEEP + *P*_insp_) [15, 16]. Here, *V*_t_ represents tidal volume, RR stands for respiratory rate, PEEP denotes positive end-expiratory pressure, and *P*_insp_ represents difference between peak inspiratory pressure and PEEP.

The primary outcome of the study was to evaluate clinical effect of MP, specifically by measuring the number of ventilator-free days at 28 days (VFD28). This composite outcome measure provides assessment for the heterogenous patients with invasive mechanical ventilators, considering both the pulmonary organ failure and mortality [17]. To account for variation in the address the variability in patients’ clinical courses, we analyzed utilized the PaO_2_/FiO_2_ ratio (P/F) as an intermediary indicator. The P/F ratio is a key marker that reflecting the clinical progression of ICU patients. It serves as a surrogate for disease development and injury, including the impact of VILI.

### Causal effect estimation and interpretability

Utilizing a directed acyclic graph (DAG) that incorporated clinical experience, the estimation process comprehensively considered the causal structure, a perspective not always attainable from data alone. This DAG facilitated the identification of potential confounding factors impacting the relationship between MP and patient outcomes (VFD28 and P/F). To estimate the causal impact of MP on patient outcomes, we used backdoor linear regression and double machine learning (DML) to estimate average treatment effects (ATEs), addressing complexities and controlling for confounding variables. Backdoor linear regression isolates the causal relationship through alternative pathways, ensuring a reliable estimate of the treatment effects of MP on patient outcomes while accommodating influencing factors. DML models, chosen for their efficacy with high-dimensional confounders or non-linear relationships, further enhance the precision of estimating the treatment effects of MP on patient outcomes [18].

To enhance the interpretability of causal model, simulation and visualization techniques were employed using dynamic DML approach. Sub-group analysis among patient population was performed to identify the conditional average treatment effects (CATEs). To facilitate the application and clinical utilization, we further developed an interactive web-based application (see supplement).

### Data processing and statistical analysis

Continuous variables with a normal distribution were presented as mean ± standard deviation and compared using Student’s *t*-test. Non-normally distributed variables were expressed as median (interquartile range, IQR) and compared using the Mann–Whitney *U* test or Kruskal–Wallis *H* test. Categorical variables were presented as numbers (percentages) and compared using the Chi-square test. The data analysis was performed using R and Python. Specific packages such as doWhy [19, 20], EconML, and Shinyapp were employed for the estimation of causal effects and visualization of results.

## Results

### Population description

Our retrospective analysis included 11,110 unique admissions receiving IMV. There are 6391 (58.3%) admitted for surgical indications. The most common indication for surgical and medical indications are cardiothoracic surgery 4403 (40.1%) and shock 3904 (35.6%), respectively. There are 7539 (67.7%) patients more than 60 years old (see Table 1, supplemental Table 1).

**Table 1:** Patient characteristic in the first 24 h of admission

	Overall *N* = 10,971	Medical *N* = 4580	Surgical *N* = 6391	*p*-value
Mean arterial pressure, mean (SD)	75.7 (16.7)	77.3 (22.7)	74.5 (10.2)	0.725
PaO_2_, mean (SD)	119.3 (37.0)	117.5 (36.3)	120.5 (37.4)	<0.001
SpO_2_, mean (SD)	97.8 (2.3)	97.6 (2.8)	97.9 (1.9)	<0.001
FiO_2_, mean (SD)	47.7 (11.0)	50.6 (12.3)	45.6 (9.4)	<0.001
Temperature, mean (SD)	35.8 (1.2)	35.3 (1.6)	36.1 (0.7)	<0.001
APACHE II, mean (SD)	19.5 (7.2)	22.7 (7.6)	17.2 (5.9)	<0.001
Age score, *n* (%)				
18–39	857 (7.8)	429 (9.4)	428 (6.7)	<0.001
40–49	851 (7.8)	431 (9.4)	420 (6.6)	
50–59	1807 (16.5)	808 (17.6)	999 (15.6)	
60–69	3141 (28.6)	1205 (26.3)	1936 (30.3)	
70–79	3213 (29.3)	1229 (26.8)	1984 (31.0)	
80+	1102 (10.0)	478 (10.4)	624 (9.8)	
Ventilator related				
Mechanical power, mean (SD)	17.7 (10.0)	21.5 (11.0)	15.0 (8.2)	<0.001
Respiratory rate, mean (SD)	17.4 (4.1)	18.8 (4.5)	16.5 (3.5)	<0.001
Tidal volume, mean (SD)	480.9 (85.1)	479.1 (80.4)	482.2 (88.2)	0.054
Minute ventilation, mean (SD)	8.6 (3.8)	9.0 (2.9)	8.3 (4.2)	<0.001
Peep, mean (SD)	7.6 (3.1)	8.8 (3.6)	6.8 (2.5)	<0.001
Peak pressure, mean (SD)	21.1 (6.3)	24.0 (6.9)	19.0 (4.9)	<0.001
Driving pressure, mean (SD)	13.0 (4.2)	14.7 (4.6)	11.7 (3.3)	<0.001

The mean MP in the first 24 h was significantly higher in the medical cohort (mean: 21.5 ± SD: 11.0 J/min) compared to the surgical cohort (15.0 ± 8.2) (*P* < 0.001). Similarly, the respiratory rate was observed to be higher in the medical cohort (18.8 ± 4.5) than in the surgical cohort (16.5 ± 3.5) (*P* < 0.001). Furthermore, the medical cohort exhibited higher mean values for PEEP (8.8 ± 3.6) compared to the surgical cohort (6.8 ± 2.5) (*P* < 0.001), and mean driving pressure was significantly higher in the medical cohort (14.7 ± 5.1) compared to the surgical cohort (11.7 ± 3.3) (*P* < 0.001), reflects different underlying disease pathologies. The intubation duration was significantly longer in the medical cohort (87.79 ± 141.25 h) compared to the surgical cohort (62.93 ± 92.45) (*P* < 0.001).

### Directed acyclic graph (DAG) description

The DAG in the Fig. 1 elucidates the interrelations between the treatment variable "mechanical_power" and the outcome variables "vent_free_days" and "pf_ratio". Notably, the DAG is directed, implying a unidirectional influence, whereby the exposure solely affects the outcomes and not vice versa. The acyclic nature of the graph further ensures the absence of feedback loops, thereby precluding the possibility of outcomes impacting the treatment. The DAG serves as a causal representation of the relationships under examination, delineating the causal effect of the mechanical power on the outcome of interest.

The estimation of the causal effect necessitates the consideration of potential confounding variables. In this study, we have identified several common clinical variables, including white blood cell (WBC) count, body temperature, pH, mean arterial blood pressure (MAP) and PaCO_2_, as a surrogate for dead space ventilation, these variables exert an influence on the patient’s disease score, subsequently impacting the intermediatory outcome "pf_ratio", which further affect ventilator-free days. The exposure variable, "mechanical_power", is directly influenced by respiratory rate, peak pressure, and lung compliance, which need to be accounted for in the causal analysis.

In the context of the DAG, both observed and unobserved confounders play a crucial role in comprehending the underlying causal mechanisms. The DAG illustrates the observed confounders through colored nodes, while unobserved confounders are represented in grey (Fig. 1). A comprehensive identification of all potential confounders, whether observable or latent, facilitates the implementation of an algorithmic approach to control for their effects in the statistical model. By doing so, we can isolate and accurately quantify the true causal effect of "mechanical_power" on the outcomes "vent_free_days" and "pf_ratio".

### Causal effect of median MP on PaO_2_/FiO_2_ ratio (P/F)

In order to assess the clinical variability of the patient, we use P/F as intermediatory outcome, which account for clinical progression such as VILI. We utilize hourly recorded variables according to DAGs that encompass all observable confounding factors. The distribution of P/F was similar in both medical and surgical populations, with a median of 216.27 (interquartile range [IQR]: 172.49–274.86) and 257.56 (IQR: 201.79–331.04), respectively.

The analysis involved 1362 patients intubated for at least 72 h. Of these, 929 were medical cases and 433 were surgical cases. Using dynamic DML methodology, the estimated ATE for the median MP in the P/F was −1.36 (95% CI: −30.62 to 27.89).

In the medical patient population, the CATE is 0.612 (95% CI: −21.155 to 22.379) while 1.152 (95% CI: −20.16 to 23.184) in surgical population. Notably, no statistically significant CATEs were observed in any other sub-cohorts (Supplemental Table 7).

### Causal effect of MP on ventilator-free day at 28 days (VFD28)

**Fig. 2:**
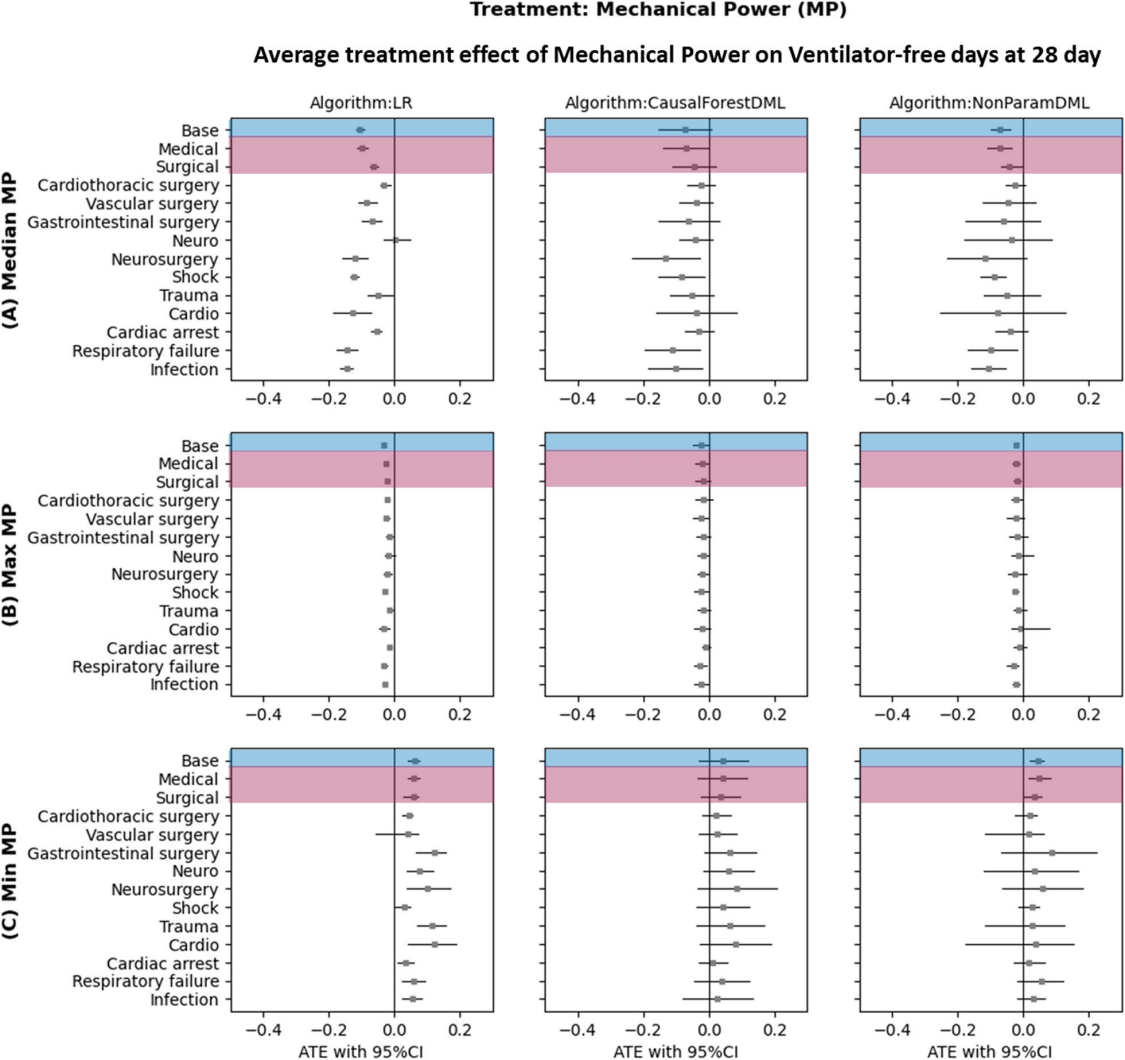
The average treatment effect (ATE) of mechanical power (MP) on ventilatory-free days at day 28 (VFD28) in difference cohorts. **A** Median MP. **B** Maximum MP. **C** Minimum MP. The findings indicate that, among the three panels, only median MP has a significant impact on VFD28. The medical population experiences a more pronounced impact from MP compared to the surgical population, resulting in fewer ventilator-free days. Patients are organized into cohorts based on intubation duration, ranging from the shortest (cardiothoracic surgery) to the longest (infection). The study demonstrates a trend of increasing effect with longer intubation times. Base: all patients, *LR* linear regression, *CF-DML* causal forest double machine learning, *NP-DML* non-parametric double machine learning

The ATE estimates were obtained for three parameters: median, maximum, and minimum mechanical power on VFD28. The analysis revealed that median mechanical power had the most substantial effect on the outcome. The estimated ATEs for median mechanical power were −0.135 (95% confidence interval [CI]: −0.15 to −0.121). In contrast, the ATEs for maximum and minimum mechanical power were −0.066 (95%CI: −0.071 to −0.061) and 0.463 (95% CI: 0.422 to 0.501), respectively, as observed in backdoor linear regression. In the CF-DML, the estimated ATEs for median, maximum, and minimum mechanical power on VFD28 were −0.095 (95%CI: −0.21 to 0.02), −0.054 (95% CI: −0.097 to −0.01), and 0.243 (95% CI: 0.068 to 0.417), respectively. Similarly, in the NP-DML approach, the estimated ATEs for median, maximum, and minimum mechanical power on VFD28 were −0.099 (95% CI: −0.129 to −0.067), −0.057 (95% CI: −0.068 to −0.045), and 0.215 (95% CI: 0.14 to 0.261), respectively (Fig. 2).

To assess the varying effects of MP across different patient populations, we conducted subgroup analyses for patients admitted to ICUs with distinct indications. The estimated conditional average treatment effect (CATE) of median MP on VFD28 was found to be more pronounced in the medical subgroup compared to the surgical subgroup. In the medical subgroup, the CATEs obtained from backdoor linear regression were −0.173 (95% CI: −0.197 to −0.143), whereas in the surgical subgroup, they were −0.072 (95% CI: −0.087 to −0.055). Employing the CF-DML method, the CATEs in the medical subgroup were −0.14 (95% CI: −0.281 to 0.001), while in the surgical subgroup, they were −0.051 (95% CI: −0.135 to 0.033). Similarly, utilizing NP-DML, the CATEs in the medical subgroup were −0.144 (95% CI: −0.198 to −0.085), and in the surgical subgroup, they were −0.055 (95% CI: −0.093 to −0.023) (Fig. 2).

Among patients with different admission diagnoses, the CATE of median MP exhibited variations, with some subgroups showing higher impacts than others. Specifically, the subgroups with cardio, respiratory, and infection diagnoses had the most substantial CATEs, while the cardiothoracic surgery subgroup showed the least. In backdoor linear regression, the CATEs for cardiac, respiratory failure, infection, and cardiothoracic surgery −0.289 (95% CI: −0.370 to −0.175), −0.263 (95% CI: −0.313 to −0.203), −0.243 (95% CI: −0.276 to −0.203), and −0.029 (95% CI: −0.040 to −0.018), respectively. In the CF-DML algorithm, the CATEs were −0.179 (95% CI: −0.359 to 0.002), −0.187 (95% CI: −0.361 to −0.013), −0.189 (95% CI: −0.344 to −0.033), and −0.018 (95% CI: −0.075 to 0.039), respectively. In NP-DML algorithm, the CATEs were −0.175 (95% CI: −0.366 to 0.223), −0.175 (95% CI: −0.256 to −0.010), −0.166 (95% CI: −0.219 to −0.057), and −0.016 (95% CI: −0.049 to 0.012), respectively (Fig. 2).

When analyzing patients admitted primarily with a diagnosis of respiratory failure, stratified by their PF ratio, we noted CATEs to be significant −0.16 (95%CI: −0.25 to −0.06) for PF ratio 200–300 and −0.104 (95%CI: −0.18 to −0.03) for PF ratio < 200 (Supplemental Table 8).

### Interpretable causal effect of mechanical power using simulation

The impact of driving pressure on P/F is distinctly evident when considering different cohorts (Supplemental Fig. 4). For individuals with a lower driving pressure (10–15 cmH_2_O), both medical and surgical groups exhibit a similar CATE. Conversely, for patients with a higher driving pressure (25–40), the absolute value of CATE escalates concordant with rising MP. Notably, this trend is more pronounced among medical patients compared to their surgical counterparts, underscoring the significance of driving pressure in influencing treatment outcomes varies across diverse clinical contexts.

In the additional analyses, we examined the independent effects of RR and *P*_insp_ on the CATE of MP for the PaO_2_/FiO_2_ ratio. As shown in Supplemental Figs. 4b and 4c, higher RR and *P*_insp_ levels both trend toward more negative CATE values as MP increases, suggesting that elevated RR and *P*_insp_ may independently contribute to increased lung injury risk.

**Fig. 3:**
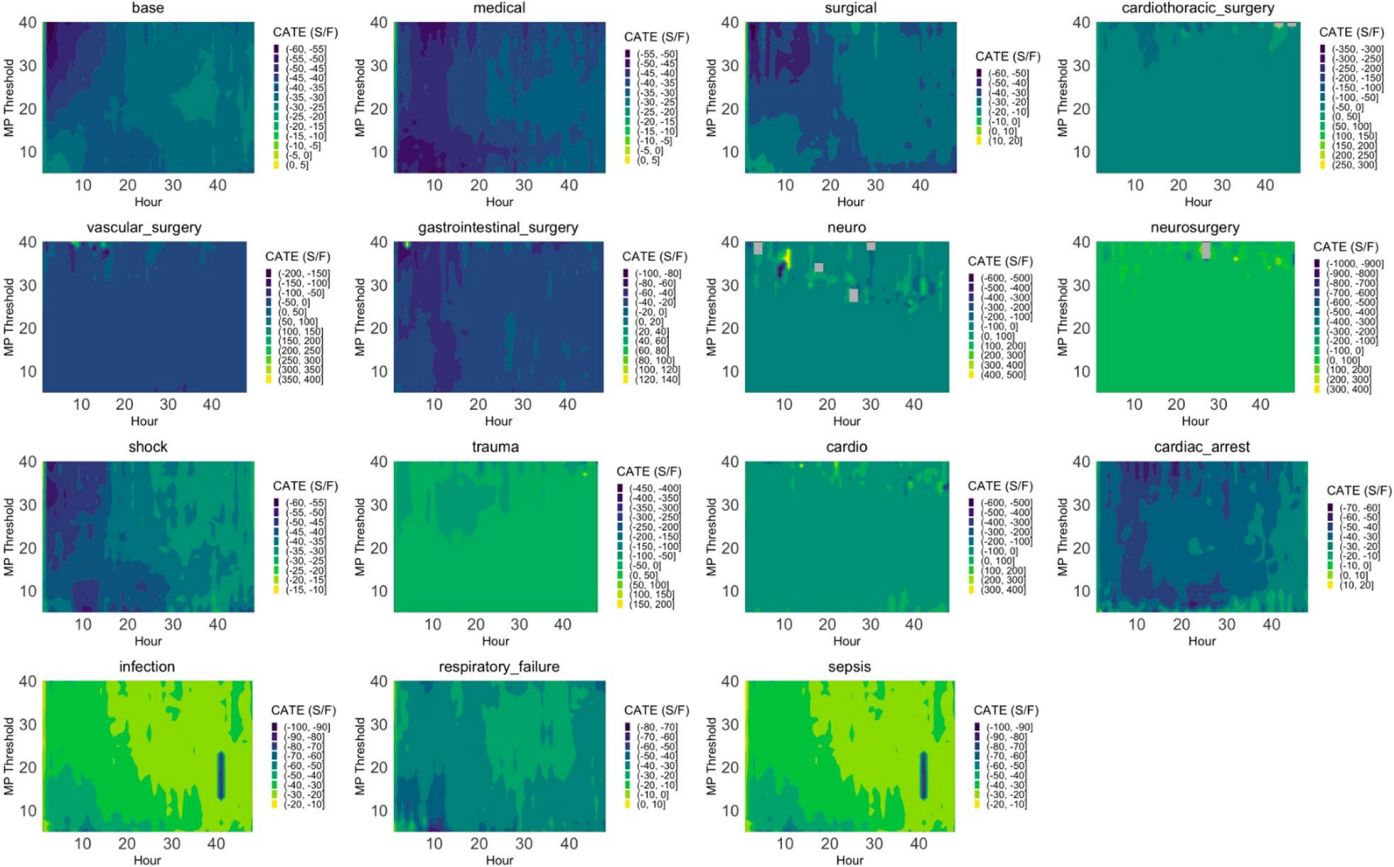
The figure presents a comprehensive series of heatmaps illustrating the CATE of MP on patient outcomes across different cohorts over time. Each heatmap corresponds to a specific patient group or condition in the context of mechanical ventilation. The x-axis represents the hours after intubation (0–40 h), while the y-axis denotes the MP thresholds (0–40 J/min). The color gradient within each heatmap reflects the CATE values, with darker colors indicating lower or negative effects and brighter colors representing higher or positive effects. The variability observed in the heatmaps underscores the complex and heterogeneous nature of patient responses to mechanical ventilation. Factors such as underlying disease conditions, individual patient characteristics, and the progression of illness influence the optimal MP threshold and its effect on outcomes

In 8640 models trained with dynamic hourly variables, distinct CATE patterns of MP threshold across patient cohorts were identified (Fig. 3). These heatmaps reveal that the impact of MP on patient outcomes is both time-dependent and cohort-specific. Rather than displaying a uniform gradient of effect from low to high MP at each time point, the patterns vary considerably among different patient groups. For example, neurosurgery and trauma patients exhibit higher CATE values at lower MP thresholds, suggesting that lower MP may be more advantageous for these cohorts. In contrast, medical and vascular surgery patients show predominantly lower CATE values across MP thresholds, indicating a different relationship between MP and outcomes.

**Fig. 4:**
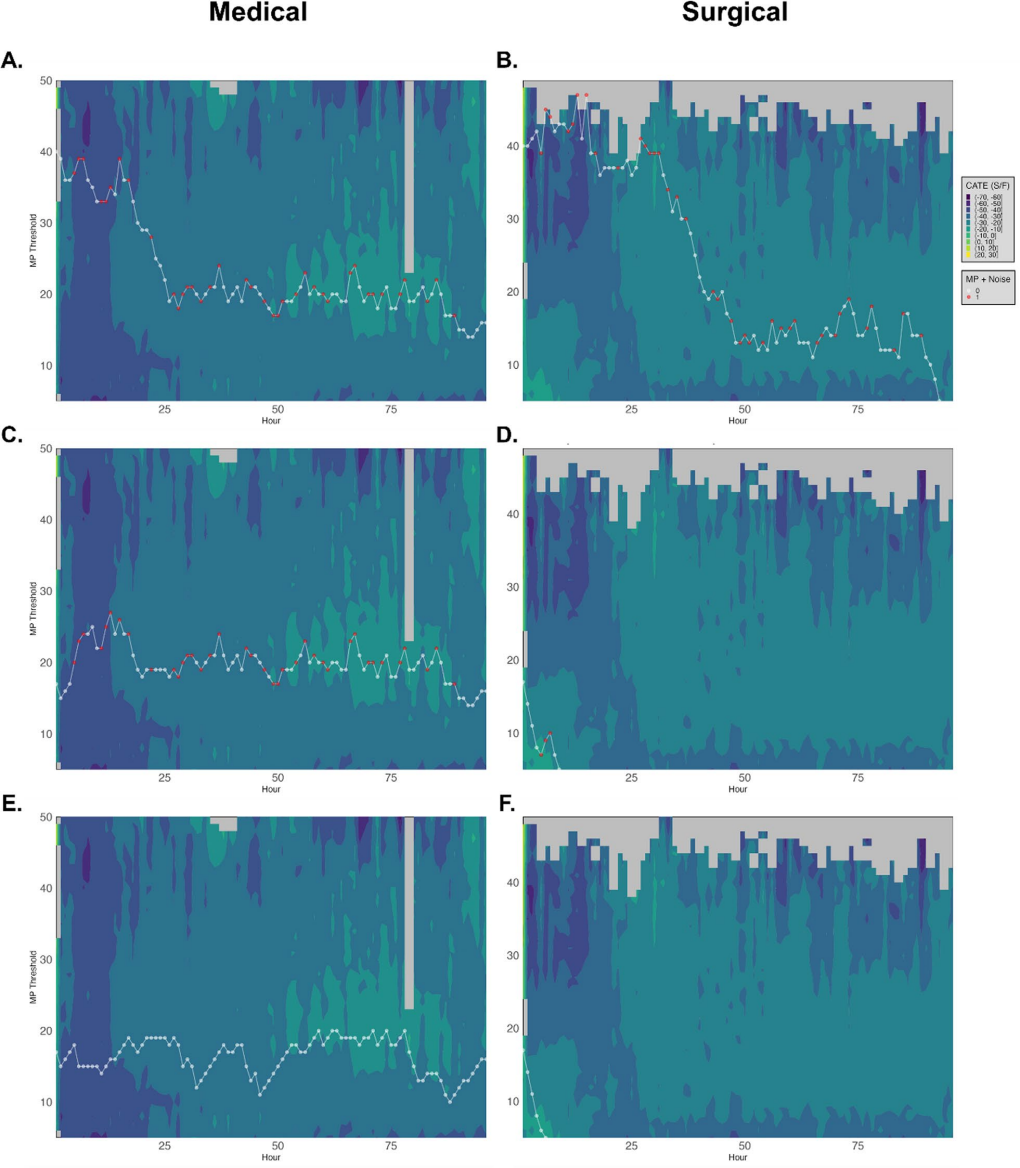
Presents simulation plots of the conditional average treatment effect (CATE) of the SpO_2_/FiO_2_ ratio over time at different mechanical power (MP) thresholds. The x-axis shows time in hours since intubation, and the y-axis represents MP thresholds in joules per minute (J/min). We adjusted the initial MP at hour 0 and introduced varying noise levels (0 to 100%) to simulate unexpected clinical events affecting ventilator settings. With a noise level of 0.4, 40% of the MP values experience random variations within predefined ranges, mimicking real-world variability. The heatmap colors correspond to the CATE calculated hourly based on the MP threshold and the previous hour’s data: brighter colors indicate higher positive CATE values (improved oxygenation), while darker colors indicate lower or negative CATE values. *White dots* represent the ideal MP trajectory without noise, and red dots indicate MP values affected by noise. Specifically: Plots **A** and **B**: initial MP threshold of 40 J/min with 40% noise. Plots **C** and **D**: initial MP threshold of 17 J/min with 40% noise. Plots **E** and **F**: initial MP threshold of 17 J/min with 0% noise (ideal scenario without variability). These simulations illustrate how different starting MP thresholds and levels of clinical variability impact expected outcomes over time. The results emphasize the importance of personalized and adaptable mechanical ventilation strategies, as optimal MP thresholds may vary based on patient-specific factors and unforeseen clinical events

To facilitate the clinical implementation of these findings, an online interactive tool was developed [21].This tool allows for the estimation and visualization of hourly CATE in both medical and surgical populations (Fig. 4). As demonstrated in the figure, for medical patients (Panels A, C, and E), brighter colors are observed at lower MP thresholds as time progresses, suggesting that reducing MP over time may improve oxygenation. For surgical patients (Panels B, D, and F), the optimal MP threshold is less consistent, with brighter colors appearing at varying MP levels and time points, indicating a more complex relationship. The optimal MP points at a specific hour are plotted as the MP that results in the minimum CATE, thus leading to minimal ventilator-induced lung injury (VILI). In Plot A, it is shown that the initial MP thresholds are 40, 39, and 37 at hours 0, 1, and 2, respectively. The optimal MP quickly drops from 40 to a range of 10–20 to minimize VILI.

## Discussion

In our study, we causatively delineated the impact of mechanical power on the P/F and the VFD28 in critically ill patients. We observed that changes in P/F and VFD28 were proportional to the administered level of MP, with more significant effects observed in disease states linked to prolonged mechanical ventilation. Intriguingly, among maximum, minimum, and median MP, the median MP exhibited the most substantial average treatment effect. We utilized a DAG, after accounting for multiple confounders, to show the intricate relationship between MP, surrogate marker easily available at bedside (P/F), and the eventual outcome (VFD28). This DAG forms the cornerstone of our work and enhances transparency of the assessment of causal inference relationships for the question under study [18]. Furthermore, our study overcomes the limitations of traditional statistical approaches that need specific thresholds of MP to dichotomize outcomes [4]. Instead, we provide simulation tools with adaptable parameters, enabling counterfactual testing. These tools also facilitate monitoring changes in MP over time at the individual patient level and facilitate tailoring of therapy to specific requirements based on the population.

Mechanical power introduced a novel approach that incorporates both pressure and volume to predict lung injury [2, 4]. However, initial studies relied on extrapolation from animal models and small control groups. Subsequently, some of the observational data were analyzed through multivariable logistic regression, to establish potential associations. For instance, a prior study demonstrated a correlation between high MP values is associated with higher 30-day mortality after accounting for confounding factors and adjusting for various medical interventions [4, 22, 23].

Randomized controlled trials investigating the clinical outcomes with mechanical power have been rarely reported. Recently, two randomized controlled trials attempted to study the effects of MP on clinical outcomes in patients with IMV [24, 25]. While the conclusions that higher mechanical power was associated with adverse outcomes were similar to our study, their sample size was much smaller and have only explored limited ICU admission diagnoses. More importantly, unlike our study, these conventional RCTs by design are limited in their ability to explore the effects of varying MP at various stages of a disease process and how that translates to clinical outcomes. Even well conducted RCTs have limitations that can influence how their results are interpreted [26]. To overcome these barriers, our study leverages the power of causal inference methods, addressing the challenges posed by non-normal distribution in real-world data and enabling meticulous control of unobserved variables, and employs DAGs to enhance transparency [27]. Moreover, our research capitalizes on the extensive patient data derived from a heterogeneous population in AmsterdamUMCdb. Through these rigorous methodologies, we have succeeded in establishing a compelling case for the causal relationship between MP and clinical outcomes in patients [18, 23].

Previous studies have often relied on models that utilize static parameters such as tidal volume, plateau pressure, or PEEP to establish associations with clinical outcomes [28–30]. However, this approach falls short in capturing the dynamic impact of mechanical power across various stages of disease and fails to account for the diverse effects of mechanical power on distinct pulmonary physiological statuses. Our study serves as a bridge to address these limitations comprehensively. By employing ATE, we gain insight into the influence of mechanical power across the entire population. Simultaneously, the use of CATE allows us to capture nuanced effects within different subgroups. While our approach advances the understanding of mechanical power’s effects, it is not without limitations. Specifically, the NP-DML and Dynamic-DML algorithms did not produce statistically significant effect on VFD28 in our study, possibly due to their sensitivity to time-varying confounders and the high variability in patient data. To accommodate the rapid evolving nature of disease progression, we establish a dynamic model [23], revealing that the mechanical power threshold is not fixed but rather varies over time. This phenomenon is demonstrated through heatmap analysis, further substantiating our study’s contribution to understanding the intricate dynamics of mechanical power’s impact. In the heatmap we constructed, each pixel represents a specific model, stratified by MP threshold and specific disease condition, for calculating the ATE both below and above the set MP threshold. Our interactive simulation application demonstrates the unique utility of using both heatmaps and ATE-guided MP targets. This approach offers advantages over relying on fixed models or thresholds, as it enhances our understanding of the real-time effects of MP and its potential clinical applicability in ventilator management.

Our findings underscore the absence of a universally applicable mechanical power threshold, reflecting the nuanced and patient-specific relationship between mechanical power and outcomes. This variability highlights the current limitation of applying mechanical power as a standardized bedside tool and emphasizes the need for individualized ventilator strategies tailored to patient-specific clinical contexts.

The strengths of our study are manifold. Firstly, we employ a robust methodology, allowing us to effectively establish causal relationships, adding a layer of depth to our findings. Secondly, our utilization of a large and heterogeneous dataset enhances the generalizability of our results, contributing to their broader applicability. Furthermore, our study’s focus on clinically relevant outcomes, such as P/F and VFD28, reinforces its significance in informing practical medical decisions. However, our study is not without its limitations. Notably, being retrospective in nature, it could be susceptible to biases. Our analysis is also constrained by data availability. For example, because patient height is not available in the AmsterdamUMCdb, we are unable to calculate the ventilatory ratio—a surrogate measure for dead space and an important confounder affecting MP—which we cannot account for in our study. Nonetheless, the inherent causal nature of our analysis affords us the ability to account for unobserved variables, emulating randomized controlled trials and thus effectively isolating the true impact of MP. Additionally, the reliance on a pre-existing database imposes constraints on the available data, potentially affecting the scope of our conclusions. It is worth noting that our model’s foundation on DAGs comes with the underlying assumptions of the current state of clinical knowledge and literature. While this anchors the study, it is important to acknowledge that DAGs can be updated with evolving insights, offering the potential to refine and update treatment effect estimations based on the latest evidence.

## Conclusion

Our study harnessed the power of causal inferential methods to elucidate the impact of MP in patients with invasive mechanical ventilation. In addition, our findings support the absence of universally applicable MP thresholds, revealing instead that these thresholds are intricately linked to individual patient lung physiology and specific disease stages. This insight was rigorously validated through comprehensive counterfactual testing. Beyond predictive machine learning, our work highlights the need for using machine learning to bridge the gap between theoretical usage of mechanical power and its bedside application. This study becomes the first step toward helping us unravel the intricate relationship between mechanical power and clinical outcomes. Further studies involving diverse population and larger sample with prospective validation are needed to enhance patient care and potentially reshape current practices.

## Supplementary Information

Additional file 1.

## Data Availability

The data analyzed in our study are available in the AmsterdamUMCdb (https://amsterdammedicaldatascience.nl/amsterdamumcdb/).

## Abbreviations

ARDS: Acute respiratory distress syndrome
ATE: Average treatment effect
CATE: Conditional average treatment effect
CF-DML: Causal forest double machine learning
COPD: Chronic obstructive pulmonary disease
DAG: Directed acyclic graph
DML: Double machine learning
FiO_2_: Fraction of inspired oxygen
ICU: Intensive care unit
MAP: Mean arterial blood pressure
MP: Mechanical power
NP-DML: Neural propensity score double machine learning
PaO_2_: Partial pressure of oxygen in arterial blood
PEEP: Positive end-expiratory pressure
*P*_insp_: Inspiratory pressure
RCT: Randomized controlled trial
RR: Respiratory rate
P/F: SpO_2_/FiO_2_ ratio
VFD28: Ventilator-free days at day 28
VILI: Ventilator-induced lung injury
*V*_t_: Tidal volume
