# Supplementary material for: Elucidating the causal relationship of mechanical power and lung injury: a dynamic approach to ventilator management
Source: Intensive Care Med Exp. 2025 Feb 28;13:28. doi: 10.1186/s40635-025-00736-w (PMC11871266; doi:10.1186/s40635-025-00736-w)
Supplement: Supplementary file 1 — Additional file 1. [file 40635_2025_736_MOESM1_ESM.docx]

Supplementary:

Elucidating the Causal Relationship of Mechanical Power and Lung Injury: A Dynamic Approach to Ventilator Management

ChaoPing Wu*^#1^, Arif Canakoglu*^#2^, Jacob Vine^3^, Anya Mathur^4^, Ronit Nath^5^, Markos Kashiouris^6^, Piyush Mathur^7^, Ari Ercole^8,9^, Paul Elbers^10^, Abhijit Duggal^1^, Ken Koon Wong*^^11^, Anirban Bhattacharyya*^^12^.

Contents

[Model Development 2](#_Toc189500426)

[Step 1: Establish DAG 2](#_Toc189500427)

[Step 2: Collapse time series - point estimate 3](#_Toc189500428)

[Step 3: Dynamic analysis with hourly data 3](#_Toc189500429)

[Step 4: Granular analysis. 3](#_Toc189500430)

[Step 5: Simulation 4](#_Toc189500431)

[Supplemental Figure 1. 6](#_Toc189500432)

[Supplemental Table 1. 6](#_Toc189500433)

[Supplemental Figure 2. 6](#_Toc189500434)

[Supplemental Figure 3. 7](#_Toc189500435)

[Supplemental Figure 4 8](#_Toc189500436)

[Ventilator analysis 10](#_Toc189500437)

[Supplemental Figure 5: 10](#_Toc189500438)

[Supplemental Table 6: 11](#_Toc189500439)

[Supplemental Table 7. 14](#_Toc189500440)

[Supplementary Table 8: 15](#_Toc189500441)

# Model Development

We executed a retrospective cohort study involving patients of all cohorts subjected to invasive mechanical ventilation (IMV), employing de-identified data sourced from AmsterdamUMCdb. Our methodological approach comprised several steps aimed at efficiently handling potential confounding variables and assessing the causal relationship between mechanical power (MP) and patient outcomes.

The formula for mechanical power (MP) is shown below:

| 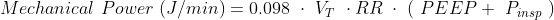  V_t_: tidal volume  RR: respiratory rate  P_insp_: Inspiratory pressure  PEEP: Positive end-expiratory pressure |
| --- |

The primary outcome of the study is to assess 28-ventilation- free days (28-VFDs) and the Spo2/Fio2 ratio (S/F). Utilization of these two outcomes is a good surrogate for ventilator-induced lung injury (VILI), which is the main concern for prolonged use of ventilation and also increasing units of mechanical power. The reason for using S/F instead of PFR is that Pao2, in general, is less frequent compared to a non-invasive method such as Spo2. The ARDS guideline has shown that S/F is a good alternative to theof PaO2/Fio2 ratio (PFR).

The study’s objective was to determine the average treatment effect (ATE) of MP on patient outcomes across diverse cohorts. Using observational study methods and the do-Operator, ATE is defined as follows:

1. **If MP is measured as an integer:** Each unit increase in MP corresponds to an expected increase or decrease in the specified outcome (either VFD or S/F), assuming MP is treated as a continuous variable.
2. **If MP is treated as binary:** The interpretation remains consistent but applies within a threshold-based framework. Refer to the supplement for interpretive examples.

| 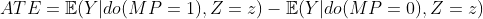  ATE: Average Treatment Effect  𝔼: Expected  Y: Outcome  MP: Mechanical Power  do: do-Operator  Z: confounders |
| --- |

We leverage the following 5 steps to evaluate ATEs:

## Step 1: Establish DAG

Initially, we constructed a directed acyclic graph (DAG) to clarify and represent the structure of potential confounders from a clinical perspective. This DAG functions as our clinical estimand, guiding the backdoor adjustment strategy and helping to avoid unintentional collider adjustments that could introduce spurious associations. Considerable effort was invested in achieving consensus on a representation that accurately reflects the causal relationship between mechanical power (MP) and patient outcomes. Using this DAG, we applied the do-Operator to MP to examine its independent relationship with outcomes. This approach surgically removed the influence of all parental nodes directly linked to MP and adjusted for severity scores using the backdoor criterion, effectively blocking any pathways that might introduce spurious associations between MP and outcomes.

## Step 2: Collapse time series - point estimate

We utilized the extensive data from AmsterdamUMCdb to extract meaningful signals by collapsing time series data into point estimates. Our analysis of the treatment-outcome relationship employed summary statistics, including mean, median, maximum, and minimum of all relevant numeric values, adjusted according to specified parameters. To estimate the average treatment effect (ATE) on the target outcome, we used the DoWhy and EconML packages DAG framework, implementing three estimation models: linear regression, Causal Forest Double Machine Learning (CF-DML), and non-parametric DML (NP-DML). Each cohort was analyzed independently due to the current lack of mixed-effect modeling capability in these packages.

The Double Machine Learning (DML) method estimates treatment effects in the presence of numerous potential confounders by leveraging machine learning algorithms alongside double robustness. DML is particularly effective in addressing high-dimensional confounding or non-parametric relationships, capturing complex associations while ensuring robust estimates—even when treatment or outcome models are not fully specified. This framework provides a flexible and reliable approach for treatment effect estimation when traditional statistical methods encounter limitations. For further details, see the DML framework overview in [source](https://academic.oup.com/ectj/article/21/1/C1/5056401).

DML estimation proceeds in two stages. In the first stage, nuisance functions are estimated using cross-fitting, where data is split into folds. Estimation occurs on one-fold while evaluation takes place on the remaining folds, reducing overfitting and improving the generalizability of nuisance function estimates. In the final stage, a conditional average treatment effect (CATE) model is estimated, integrating the nuisance functions to capture the causal effect of the treatment variable on the outcome. This CATE model generates an ATE estimate that accounts for confounding variables and covariates.

## Step 3: Dynamic analysis with hourly data

We subsequently expanded our time series data to hourly intervals to enhance signal detection, with S/F ratio as the outcome measure, expected to be recorded hourly. Missing values were addressed by carrying forward the most recent available data. This analysis employed the dynamic DML method, as supported by EconML, an extension of the traditional DML approach that accommodates treatments assigned sequentially over time. This estimator accounts for causal effects of treatments on subsequent outcomes, allowing for temporal dynamics in the analysis [source](https://arxiv.org/abs/2002.07285).

This dynamic approach enabled a more realistic and detailed examination of a patient’s disease trajectory. For each model and cohort, we estimated the ATE along with 95% confidence intervals, as this method currently lacks mixed-effect modeling capabilities [source](https://econml.azurewebsites.net/spec/estimation/dynamic_dml.html).

## Step 4: Granular analysis.

To gain a more comprehensive understanding of the model's dynamicity at a fine-grained level, we employed an iterative linear regression approach on the MP sequence, ranging from 5 to 50 with a one-unit increment per hour, using backdoor adjustment. The MP thresholds were treated as binary values (0 or 1), where a value of 1 indicated that the MP value was above the specific threshold (ranging from 5 to 50), while a value of 0 indicated it was below.

The study encompassed a timeframe of 96 hours for each cohort, with the primary outcome of interest being the assessment of S/F (not specified). The metric used for evaluation was the Average Treatment Effect (ATE) along with its corresponding 95% confidence interval. This comprehensive analysis resulted in the development of 51,840 models, achieved through the combination of 12 cohorts, 45 MP thresholds, and 96 hours of data for each cohort.

Given the significant diversity and imbalance in patient characteristics and numbers, we performed cohort pattern assessments within their respective groups. For visualization purposes, we utilized 3D mesh plots with the plotly package to examine the ATE pattern for each cohort (reference: [https://plotly.com/chart-studio-help/citations/](https://plotly.com/chart-studio-help/citations/**)). Additionally, 2D contours of the 3D surfaces were generated to offer an overarching view of all cohorts and facilitate further interpretation of the findings.

## Step 5: Simulation

To demonstrate the model's applicability and enhance its relevance to ICU patients, we conducted a simulation using the medical and surgical cohorts for our CATE-targeted mechanical power (MP) threshold analysis. We anticipated that these cohorts would provide a sufficiently large sample size to yield accurate results.

The simulation parameters included the hour after intubation (ranging from 1 to 96 hours), MP value (5–50 J/min), MP standard deviation, noise level (0–100%), and minimum and maximum noise thresholds. This simulation applied a CATE-guided approach to identify the optimal next-hour MP value, with the novel aim of serving as a "weaning" protocol to reduce the risk of ventilator-induced lung injury (VILI).

The MP standard deviation parameter allowed us to set a realistic minimum MP value, defining an achievable reduction target for each subsequent hour. Noise parameters employed Monte Carlo sampling to introduce random fluctuations, simulating unpredictable clinical events that might necessitate adjustments in the current MP, thereby reflecting real-life variability. Given the simulation's dynamic nature, a web application was developed to enable users to interact with the CATE-targeted MP threshold simulation and visualize the weaning process. Although experimental, this proof-of-concept aims to assess the feasibility of an MP threshold weaning protocol, potentially setting the stage for future experimental validation. A pseudorandom seed was incorporated to ensure reproducibility in the Monte Carlo simulation's randomization.

The simulation results are presented in Figure 4 of the main manuscript, which displays a series of six heatmaps (Panels A–F) illustrating simulations of the CATE of MP on the SpO₂/FiO₂ (S/F) ratio over time in medical and surgical patients receiving mechanical ventilation. The heatmaps are arranged in a 3×2 grid, with medical patient simulations on the left (Panels A, C, and E) and surgical patient simulations on the right (Panels B, D, and F).

In these heatmaps, the x-axis represents time after intubation (0–100 hours), indicating the progression since the initiation of mechanical ventilation, while the y-axis denotes the MP thresholds (0–50 J/min), representing potential target MP settings for the next hour. The color gradient within each heatmap reflects the CATE values for targeting a specific MP at a particular time point: brighter colors indicate higher positive CATE values (suggesting improved oxygenation), and darker colors indicate lower or negative CATE values (suggesting worsened oxygenation).

The simulations incorporated different initial MP thresholds and levels of clinical variability (noise). Panels A and B started with an initial MP threshold of 40 J/min, while Panels C–F started with 17 J/min. Panels A–D included a 40% noise factor to represent real-world variability and unpredicted clinical events that necessitate adjustments in ventilator settings; in these panels, red dots represent the actual MP applied to the patient, affected by clinical variability. Panels E and F were simulations without noise, representing an ideal scenario with consistent ventilator settings, with white dots representing the ideal MP trajectory unaffected by clinical variability.

The impact of clinical variability is evident, as the presence of noise (Panels A–D) demonstrates how unpredicted clinical events can cause deviations from the ideal MP trajectory (red dots diverging from white dots). This variability necessitates continuous reassessment and adjustment of ventilation strategies to optimize patient outcomes. Additionally, starting with a lower initial MP threshold of 17 J/min (Panels C–F) generally results in more positive CATE values over time compared to starting at 40 J/min (Panels A and B), suggesting that initiating mechanical ventilation with lower MP settings may be beneficial, particularly for medical patients.

Comparing patient cohorts, medical patients show a clearer trend toward improved outcomes with lower MP thresholds over time, while surgical patients display greater variability in optimal MP thresholds, indicating that factors unique to surgical patients may influence their response to mechanical ventilation.

The clinical implications of these findings underscore the importance of personalized ventilation strategies. The simulations highlight the necessity of tailoring mechanical ventilation settings to individual patient needs, accounting for patient type (medical vs. surgical), time since intubation, and real-time clinical factors. A one-size-fits-all approach to MP thresholds may not be optimal, and dynamic adjustments based on continuous monitoring could enhance patient outcomes. The variability and time-dependent changes observed in the heatmaps emphasize the need for clinicians to frequently reassess ventilator settings. Incorporating tools that can predict the CATE of MP adjustments may assist clinicians in making informed decisions.

The main objective of our analysis was to determine the CATE of MP on outcomes across patient cohorts. We employed the DoWhy library for causal inference and EconML for econometric causal effect estimation using non-parametric double machine learning. To identify an optimal MP threshold, we used a machine learning approach, training models at various MP levels for comparative analysis. We assessed the robustness of our findings through refutation estimation, which tests the reliability of causal estimates. This comprehensive, dynamic methodology provided deeper insights into the ATE of MP, thereby offering a nuanced perspective on MP's clinical implications across different patient cohorts and stages of disease.

# Supplemental Figure 1.

Patient diagram


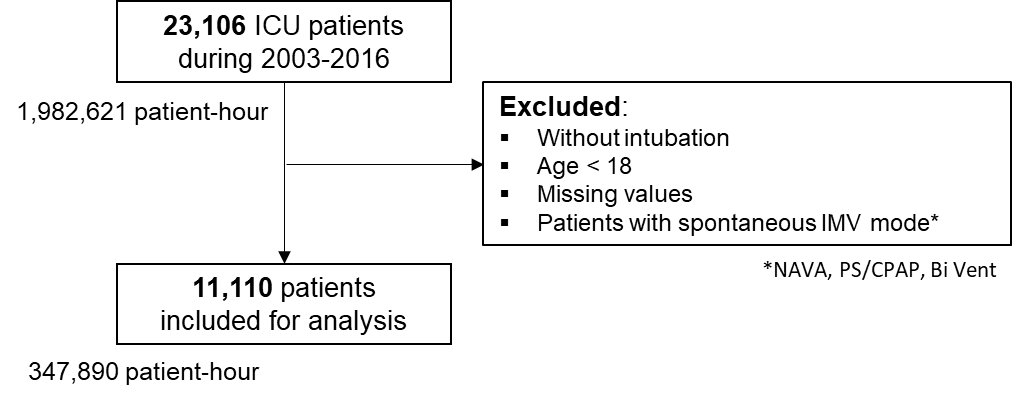


# Supplemental Table 1.

This table presents a subgroup analysis of ICU admission reasons with associated patient counts and mean intubation durations. Surgical admissions comprised 58.3%. Patients could have multiple admission categories (e.g., surgical and cardiothoracic surgery), reflecting overlapping clinical needs.

| Admission reason | Number of patients | Percentage |
| --- | --- | --- |
| is_surgical | 6391 | 58.3% |
| is_cardiothoracic_surgery | 4403 | 40.1% |
| is_respiratory_failure | 697 | 6.4% |
| is_neurosurgery | 465 | 4.2% |
| is_gastrointestinal_surgery | 463 | 4.2% |
| is_cardiac_arrest | 863 | 7.9% |
| is_vascular_surgery | 560 | 5.1% |
| is_trauma | 532 | 4.8% |
| is_neuro | 240 | 2.2% |
| is_cardio | 237 | 2.2% |
| is_infection | 1650 | 15.0% |
| is_shock | 3904 | 35.6% |
| is_sepsis | 1642 | 15.0% |

# Supplemental Figure 2.

Shows simulation with percent noise set at 50% with *Uniform*(minimum 1, maximum 5). Red diamonds represent MP with added random noise.


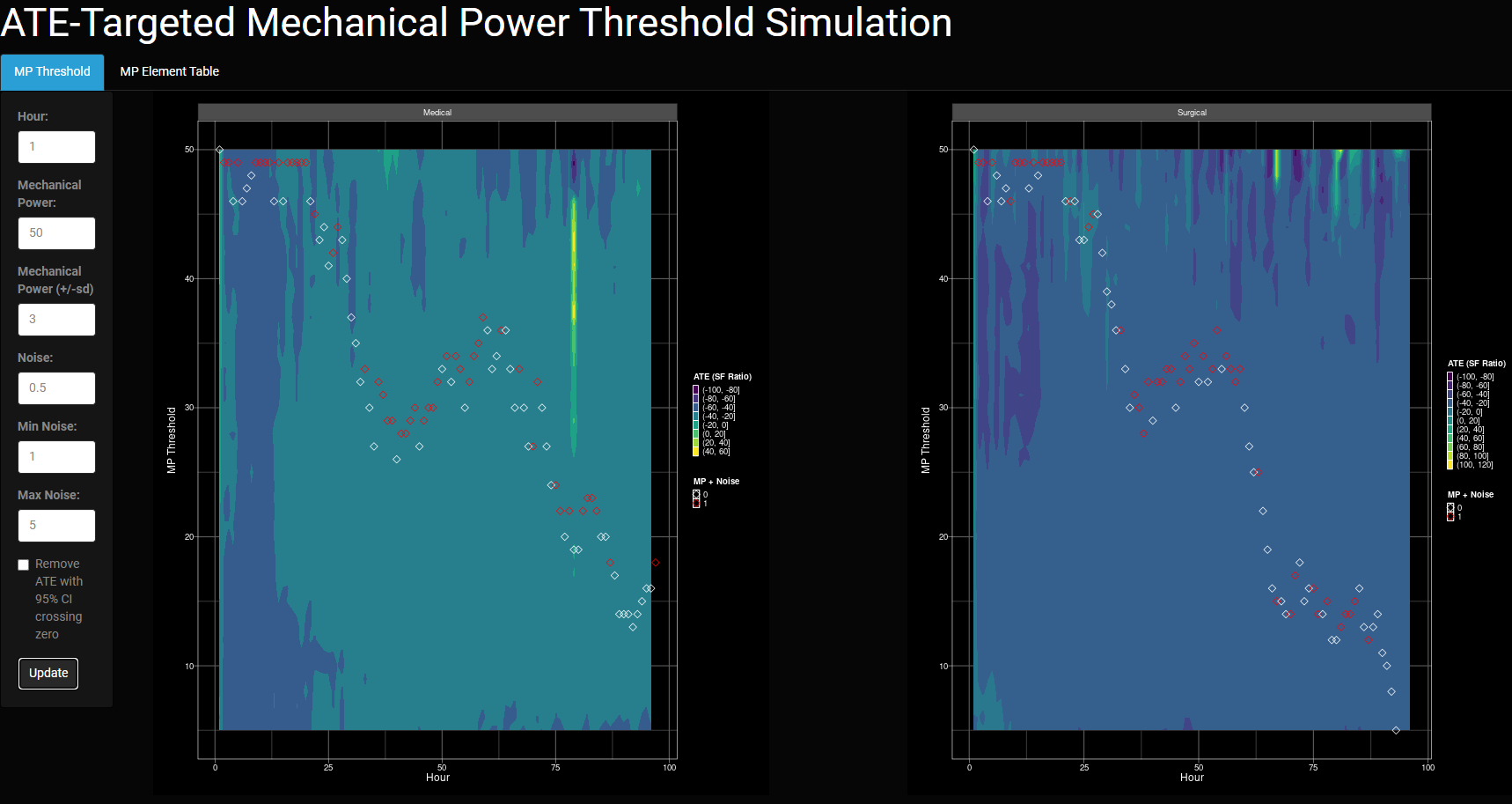


# Supplemental Figure 3.

Shows direct conditional query of a specific MP to further assess which parameters would be best adjusted based on ATE. For example, when an MP of 30 in medical cohort is thought to be the next target, on the 5th hour, with fixed PEEP of 15 (due to medical condition), adjustment of respiratory rate, tidal volume, and inspiratory pressure are the only parameters that can be tuned. This table shows the possible range of tuning options sorted descending through the best ATE.


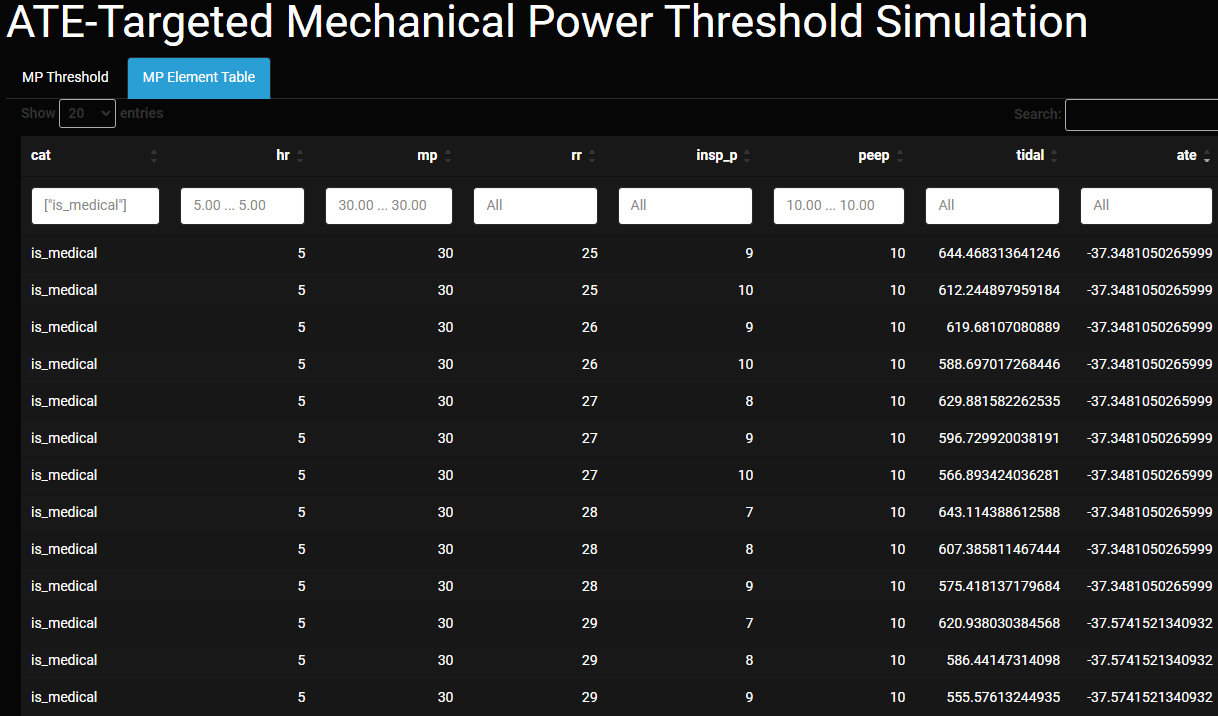


# Supplemental Figure 4

**Interpretable Causal Effect of Mechanical Power Using Simulation**


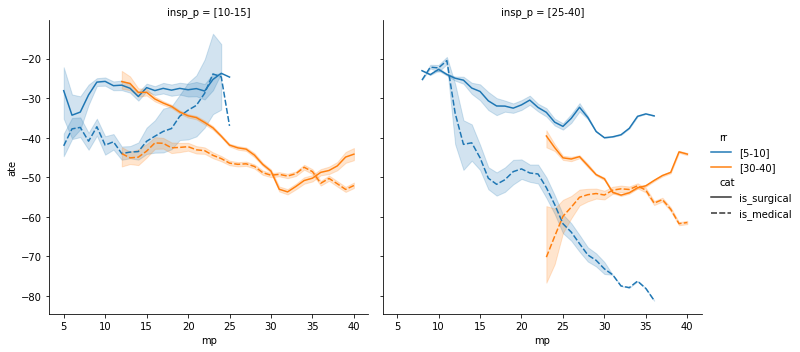


**Figure 4-a**: The impact of driving pressure on patient outcomes is distinctly evident when considering different cohorts. For individuals with a lower driving pressure (10-15), both medical and surgical groups exhibit a similar average treatment effect (ATE), remaining consistent despite variations in mechanical power (MP). Conversely, for patients with a higher driving pressure (25-40), the absolute value of ATE escalates alongside rising MP. Notably, this trend is more pronounced among medical patients compared to their surgical counterparts, underscoring the significance of driving pressure in influencing treatment outcomes varies across diverse clinical contexts.


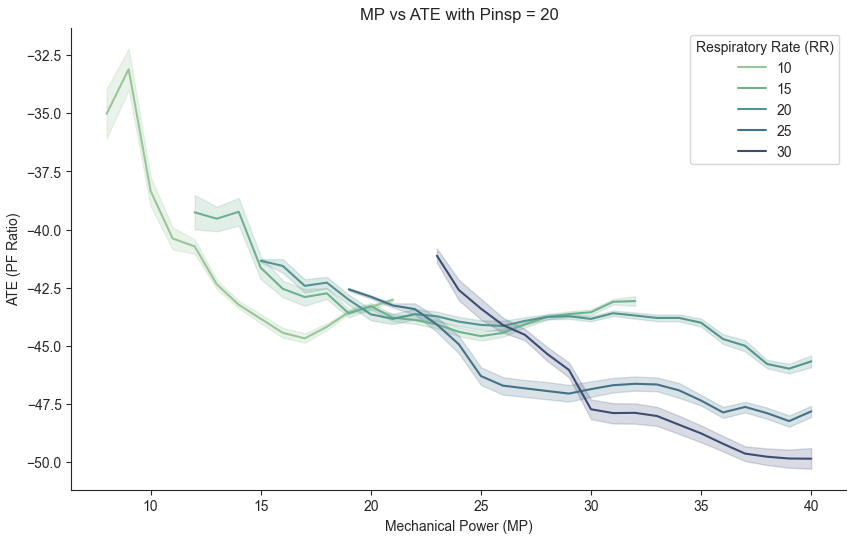


**Figure 4-b**: Effect of Varying Respiratory Rate on the Conditional Average Treatment Effect (CATE) of Mechanical Power (MP) for the PaO₂/FiO₂ Ratio with Constant Pinsp.

This figure illustrates the relationship between MP and the CATE of the PF ratio across different RR values while holding Pinsp constant at 20 cm H₂O. The x-axis represents MP, and the y-axis denotes the CATE in terms of the PF ratio. Lines correspond to various RR values (10–30 breaths per minute). As RR increases, there is an observed trend toward more negative CATE values with rising MP, suggesting that higher RR settings may amplify the adverse effects of MP on lung function due to increased mechanical strain and ventilation demands.
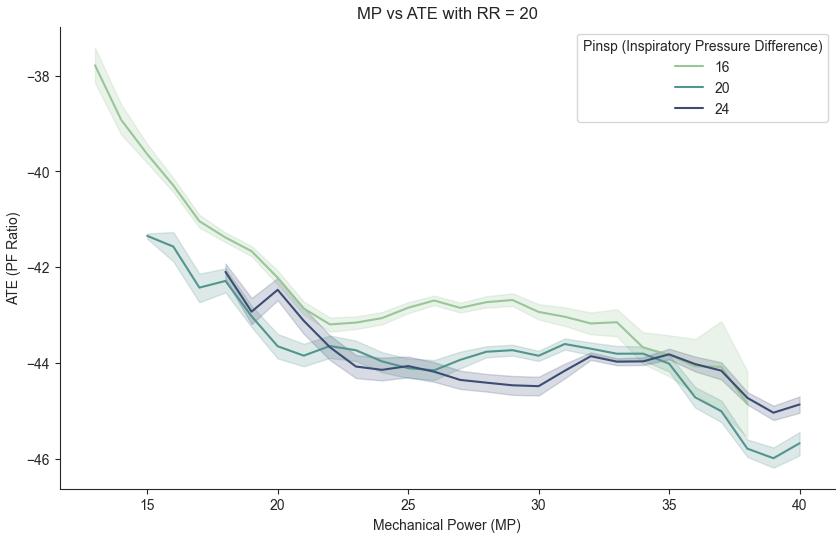


**Figure 4c.** *Effect of Varying Inspiratory Pressure on the CATE of Mechanical Power (MP) for the PF Ratio with Constant RR.*

The figure shows the relationship between MP and the CATE of the PF ratio for varying levels of Pinsp while holding RR constant at 20 breaths per minute. The x-axis represents MP, and the y-axis represents the CATE in terms of the PF ratio. Lines correspond to different Pinsp values (16–25 cm H₂O). Although the curves for Pinsp = 20 and Pinsp = 24 overlap, there is a trend toward more negative CATE values with higher Pinsp, particularly as MP increases. This suggests that elevated inspiratory pressures may contribute to a greater risk of lung injury due to increased mechanical stress on alveolar structures.

# Ventilator analysis

In this analysis, we implemented analysis in the following population:

**Supplemental Table 2**.

| **Category** | **Count** |  |
| --- | --- | --- |
| PC | 341085 | 95.59% |
| VC | 14483 | 4.06% |
| PC, VC | 1246 | 0.35% |
| Other | 17 | 0.00% |

## Supplemental Figure 5:

Directed Acyclic Graph (DAG) for Assessing the Causal Impact of Mechanical Power on spo₂/fio₂ Ratio and 28-Day Ventilation-Free Days with Adjustment for Patient and Disease Severity Variables

This DAG illustrates the relationships among various variables in evaluating the ATE of mechanical power on the S/F ratio and VFD28. The DAG outlines the causal pathways and specifies the confounders adjusted for in this analysis. Key variables include patient-specific factors (e.g., age, sex, past medical history), disease severity indicators (e.g., creatinine, bilirubin, sodium, hematocrit, temperature, and FiO₂), and physiological metrics related to mechanical ventilation (e.g., peak pressure, respiratory rate, compliance, delta V, PEEP, and inspiratory pressure).

These pathways inform the potential causal effect of mechanical power on the outcomes while accounting for complex interactions across multiple physiological domains, thereby enhancing the precision of the ATE estimation.

## Supplemental Table 6:

**sTable 6-1** the effect of Median MP on VFD28

| Population | CATE (95% CI) | algorithm |
| --- | --- | --- |
| Overall | -0.135 (-0.15 to -0.121) | LR |
| Overall | -0.095 (-0.21 to 0.02) | CausalForestDML |
| Overall | -0.099 (-0.129 to -0.067) | NonParamDML |
| is_surgical | -0.072 (-0.087 to -0.055) | LR |
| is_surgical | -0.051 (-0.135 to 0.033) | CausalForestDML |
| is_surgical | -0.055 (-0.093 to -0.023) | NonParamDML |
| is_medical | -0.173 (-0.197 to -0.143) | LR |
| is_medical | -0.14 (-0.281 to 0.001) | CausalForestDML |
| is_medical | -0.144 (-0.198 to -0.085) | NonParamDML |
| is_cardiothoracic_surgery | -0.029 (-0.04 to -0.018) | LR |
| is_cardiothoracic_surgery | -0.018 (-0.075 to 0.039) | CausalForestDML |
| is_cardiothoracic_surgery | -0.016 (-0.049 to 0.012) | NonParamDML |
| is_respiratory_failure | -0.263 (-0.313 to -0.203) | LR |
| is_respiratory_failure | -0.187 (-0.361 to -0.013) | CausalForestDML |
| is_respiratory_failure | -0.175 (-0.256 to -0.01) | NonParamDML |
| is_neurosurgery | -0.122 (-0.168 to -0.064) | LR |
| is_neurosurgery | -0.111 (-0.254 to 0.032) | CausalForestDML |
| is_neurosurgery | -0.097 (-0.217 to 0.077) | NonParamDML |
| is_gastrointestinal_surgery | -0.163 (-0.22 to -0.103) | LR |
| is_gastrointestinal_surgery | -0.103 (-0.24 to 0.033) | CausalForestDML |
| is_gastrointestinal_surgery | -0.109 (-0.252 to 0.045) | NonParamDML |
| is_cardiac_arrest | -0.072 (-0.11 to -0.033) | LR |
| is_cardiac_arrest | -0.077 (-0.175 to 0.022) | CausalForestDML |
| is_cardiac_arrest | -0.082 (-0.173 to 0.012) | NonParamDML |
| is_vascular_surgery | -0.145 (-0.232 to -0.083) | LR |
| is_vascular_surgery | -0.047 (-0.143 to 0.05) | CausalForestDML |
| is_vascular_surgery | -0.059 (-0.143 to 0.069) | NonParamDML |
| is_trauma | -0.194 (-0.235 to -0.154) | LR |
| is_trauma | -0.132 (-0.267 to 0.003) | CausalForestDML |
| is_trauma | -0.158 (-0.305 to -0.032) | NonParamDML |
| is_neuro | -0.059 (-0.118 to 0.032) | LR |
| is_neuro | -0.081 (-0.214 to 0.051) | CausalForestDML |
| is_neuro | -0.115 (-0.363 to 0.028) | NonParamDML |
| is_cardio | -0.289 (-0.37 to -0.175) | LR |
| is_cardio | -0.179 (-0.359 to 0.002) | CausalForestDML |
| is_cardio | -0.175 (-0.366 to 0.223) | NonParamDML |
| is_infection | -0.243 (-0.276 to -0.203) | LR |
| is_infection | -0.189 (-0.344 to -0.033) | CausalForestDML |
| is_infection | -0.166 (-0.219 to -0.057) | NonParamDML |
| is_shock | -0.189 (-0.209 to -0.163) | LR |
| is_shock | -0.125 (-0.26 to 0.009) | CausalForestDML |
| is_shock | -0.125 (-0.174 to -0.075) | NonParamDML |
| is_sepsis | -0.241 (-0.272 to -0.202) | LR |
| is_sepsis | -0.162 (-0.317 to -0.007) | CausalForestDML |
| is_sepsis | -0.182 (-0.245 to -0.099) | NonParamDML |

**sTable 6-2** the effect of Maximum MP on VFD28

| **Population** | **CATE (95% CI)** | **algorithm** |
| --- | --- | --- |
| Overall | -0.066 (-0.071 to -0.061) | LR |
| Overall | -0.054 (-0.097 to -0.01) | CausalForestDML |
| Overall | -0.057 (-0.068 to -0.045) | NonParamDML |
| is_surgical | -0.047 (-0.054 to -0.042) | LR |
| is_surgical | -0.045 (-0.088 to -0.002) | CausalForestDML |
| is_surgical | -0.049 (-0.062 to -0.038) | NonParamDML |
| is_medical | -0.062 (-0.067 to -0.055) | LR |
| is_medical | -0.049 (-0.087 to -0.01) | CausalForestDML |
| is_medical | -0.051 (-0.068 to -0.029) | NonParamDML |
| is_cardiothoracic_surgery | -0.037 (-0.045 to -0.028) | LR |
| is_cardiothoracic_surgery | -0.035 (-0.078 to 0.008) | CausalForestDML |
| is_cardiothoracic_surgery | -0.042 (-0.069 to -0.018) | NonParamDML |
| is_respiratory_failure | -0.07 (-0.08 to -0.057) | LR |
| is_respiratory_failure | -0.062 (-0.099 to -0.026) | CausalForestDML |
| is_respiratory_failure | -0.056 (-0.075 to -0.021) | NonParamDML |
| is_neurosurgery | -0.044 (-0.056 to -0.031) | LR |
| is_neurosurgery | -0.052 (-0.084 to -0.021) | CausalForestDML |
| is_neurosurgery | -0.043 (-0.066 to -0.012) | NonParamDML |
| is_gastrointestinal_surgery | -0.057 (-0.075 to -0.03) | LR |
| is_gastrointestinal_surgery | -0.053 (-0.095 to -0.012) | CausalForestDML |
| is_gastrointestinal_surgery | -0.052 (-0.085 to -0.006) | NonParamDML |
| is_cardiac_arrest | -0.038 (-0.046 to -0.028) | LR |
| is_cardiac_arrest | -0.025 (-0.05 to 0) | CausalForestDML |
| is_cardiac_arrest | -0.032 (-0.061 to -0.002) | NonParamDML |
| is_vascular_surgery | -0.057 (-0.07 to -0.04) | LR |
| is_vascular_surgery | -0.057 (-0.097 to -0.018) | CausalForestDML |
| is_vascular_surgery | -0.052 (-0.086 to -0.005) | NonParamDML |
| is_trauma | -0.04 (-0.056 to -0.022) | LR |
| is_trauma | -0.041 (-0.076 to -0.006) | CausalForestDML |
| is_trauma | -0.042 (-0.076 to -0.005) | NonParamDML |
| is_neuro | -0.026 (-0.044 to 0.001) | LR |
| is_neuro | -0.017 (-0.073 to 0.039) | CausalForestDML |
| is_neuro | -0.04 (-0.102 to 0.025) | NonParamDML |
| is_cardio | -0.081 (-0.103 to -0.056) | LR |
| is_cardio | -0.068 (-0.11 to -0.027) | CausalForestDML |
| is_cardio | -0.077 (-0.158 to 0.006) | NonParamDML |
| is_infection | -0.07 (-0.077 to -0.06) | LR |
| is_infection | -0.054 (-0.09 to -0.017) | CausalForestDML |
| is_infection | -0.056 (-0.071 to -0.04) | NonParamDML |
| is_shock | -0.073 (-0.08 to -0.067) | LR |
| is_shock | -0.058 (-0.1 to -0.016) | CausalForestDML |
| is_shock | -0.057 (-0.071 to -0.034) | NonParamDML |
| is_sepsis | -0.07 (-0.077 to -0.06) | LR |
| is_sepsis | -0.054 (-0.09 to -0.018) | CausalForestDML |
| is_sepsis | -0.052 (-0.065 to -0.028) | NonParamDML |

**sTable 6-3** the effect of Minimum MP on VFD28

| **Population** | **CATE (95% CI)** | **algorthm** |
| --- | --- | --- |
| Overall | 0.463 (0.422 to 0.501) | LR |
| Overall | 0.243 (0.068 to 0.417) | CausalForestDML |
| Overall | 0.215 (0.14 to 0.261) | NonParamDML |
| is_surgical | 0.376 (0.321 to 0.421) | LR |
| is_surgical | 0.163 (0.028 to 0.299) | CausalForestDML |
| is_surgical | 0.163 (0.083 to 0.232) | NonParamDML |
| is_medical | 0.414 (0.356 to 0.459) | LR |
| is_medical | 0.25 (0.045 to 0.455) | CausalForestDML |
| is_medical | 0.197 (0.068 to 0.265) | NonParamDML |
| is_cardiothoracic_surgery | 0.255 (0.223 to 0.282) | LR |
| is_cardiothoracic_surgery | 0.128 (0.022 to 0.234) | CausalForestDML |
| is_cardiothoracic_surgery | 0.127 (0.069 to 0.205) | NonParamDML |
| is_respiratory_failure | 0.464 (0.354 to 0.552) | LR |
| is_respiratory_failure | 0.26 (0.013 to 0.508) | CausalForestDML |
| is_respiratory_failure | 0.24 (-0.041 to 0.375) | NonParamDML |
| is_neurosurgery | 0.433 (0.336 to 0.522) | LR |
| is_neurosurgery | 0.167 (-0.035 to 0.369) | CausalForestDML |
| is_neurosurgery | 0.219 (0.029 to 0.432) | NonParamDML |
| is_gastrointestinal_surgery | 0.494 (0.405 to 0.558) | LR |
| is_gastrointestinal_surgery | 0.215 (0.028 to 0.402) | CausalForestDML |
| is_gastrointestinal_surgery | 0.225 (0.029 to 0.425) | NonParamDML |
| is_cardiac_arrest | 0.355 (0.292 to 0.4) | LR |
| is_cardiac_arrest | 0.136 (0 to 0.273) | CausalForestDML |
| is_cardiac_arrest | 0.11 (0.002 to 0.216) | NonParamDML |
| is_vascular_surgery | 0.354 (0.132 to 0.41) | LR |
| is_vascular_surgery | 0.14 (-0.042 to 0.321) | CausalForestDML |
| is_vascular_surgery | 0.16 (-0.067 to 0.318) | NonParamDML |
| is_trauma | 0.357 (0.238 to 0.449) | LR |
| is_trauma | 0.11 (-0.086 to 0.306) | CausalForestDML |
| is_trauma | 0.136 (-0.12 to 0.328) | NonParamDML |
| is_neuro | 0.414 (0.3 to 0.501) | LR |
| is_neuro | 0.31 (0.131 to 0.49) | CausalForestDML |
| is_neuro | 0.167 (-0.131 to 0.429) | NonParamDML |
| is_cardio | 0.355 (0.062 to 0.466) | LR |
| is_cardio | 0.112 (-0.065 to 0.289) | CausalForestDML |
| is_cardio | 0.079 (-0.396 to 0.318) | NonParamDML |
| is_infection | 0.497 (0.44 to 0.554) | LR |
| is_infection | 0.311 (0.076 to 0.546) | CausalForestDML |
| is_infection | 0.251 (0.117 to 0.346) | NonParamDML |
| is_shock | 0.438 (0.348 to 0.509) | LR |
| is_shock | 0.219 (0.024 to 0.415) | CausalForestDML |
| is_shock | 0.211 (0.13 to 0.318) | NonParamDML |
| is_sepsis | 0.496 (0.429 to 0.551) | LR |
| is_sepsis | 0.306 (0.08 to 0.532) | CausalForestDML |
| is_sepsis | 0.26 (0.132 to 0.363) | NonParamDML |

# Supplemental Table 7.

**Table 7:** Conditional Average Treatment Effects (CATEs) of Mechanical Power (MP) on PaO₂/FiO₂ (P/F) Ratio Across Different Patient Cohorts: This table presents the CATE values with 95% confidence intervals for the effect of MP on the P/F ratio across various patient populations. Each row represents a distinct patient cohort, such as surgical, medical, or respiratory failure patients, to capture potential subgroup-specific effects. The base population represents the entire cohort without subgroup differentiation.

| Population | CATE (95% CI) |
| --- | --- |
| Overall | 0.866 (-1.279 to 3.01) |
| is_surgical | 1.512 (-20.16 to 23.184) |
| is_medical | 0.612 (-21.155 to 22.379) |
| is_cardiothoracic_surgery | 73.049 (-516.333 to 662.431) |
| is_respiratory_failure | -17.429 (-70.254 to 35.396) |
| is_neurosurgery | 8.52 (-169.178 to 186.219) |
| is_gastrointestinal_surgery | -3.743 (-51.878 to 44.392) |
| is_cardiac_arrest | 3.251 (-117.947 to 124.449) |
| is_vascular_surgery | 99.253 (-1142.87 to 1341.377) |
| is_trauma | 29.295 (-77.907 to 136.498) |
| is_neuro | -14.415 (-124.244 to 95.413) |
| is_cardio | -31.847 (-321.57 to 257.877) |
| is_infection | -3.572 (-26.118 to 18.973) |
| is_shock | -2.384 (-28.267 to 23.5) |

The CATE values show no statistically significant differences across most patient cohorts. This lack of statistical significance likely reflects the heterogeneity of the patient population and suggests that MP does not uniformly affect respiratory outcomes across all groups. These results emphasize the complexity of MP’s effects, potentially indicating that certain patient characteristics and underlying conditions modulate the impact of MP on lung function.

# **Supplementary Table 8**:

Subgroup Analysis of CATE in Respiratory Failure Patients by ARDS Severity

**Description:** This table presents a subgroup analysis of conditional average treatment effects (CATE) in respiratory failure patients categorized by ARDS severity, determined by PaO₂/FiO₂ ratio (PFR) thresholds: No ARDS (PFR > 300), Mild ARDS (PFR 200–300), and Moderate-to-Severe ARDS (PFR ≤ 200). The CATE values are reported for each severity group using three statistical algorithms: Linear Regression (LR), Causal Forest DML (CausalForestDML), and Nonparametric DML (NonParamDML), with corresponding 95% confidence intervals. This analysis highlights variations in treatment effects across ARDS severity levels, with more pronounced effects observed in Moderate-to-Severe ARDS as compared to No ARDS and Mild ARDS categories.

| **CATEs of algorithms (95% CI)** | **No ARDS (N = 78)** | **Mild ARDS (N=261)** | **Mod-to-Severe ARDS (N = 355)** |
| --- | --- | --- | --- |
| **LR** | -0.04 (-0.13 to 0.05) | -0.16 (-0.25 to -0.06) | -0.104 (-0.18 to -0.03) |
| **CausalForestDML** | -0.06 (-0.17 to 0.05) | -0.04 (-0.20 to 0.12) | -0.10 (-0.28 to 0.07) |
| **NonParamDML** | -0.08 (-0.26 to 0.06) | -0.11 (-0.36 to 0.10) | -0.11 (-0.26 to 0.11) |
